# Assessment of isokinetic trunk muscle strength and its association with health-related quality of life in patients with degenerative spinal deformity

**DOI:** 10.1186/s12891-020-03844-8

**Published:** 2020-12-09

**Authors:** Sen Yang, Can Chen, Shiyu Du, Yong Tang, Kai Li, Xueke Yu, Jiulin Tan, Chengmin Zhang, Zhigang Rong, Jianzhong Xu, Wenjie Wu, Fei Luo

**Affiliations:** 1 Department of Orthopaedics, Southwest Hospital, Third Military Medical University (Army Medical University), 30 Gaotanyan Street, Shapingba, Chongqing, 400038 China; 2 Department of Orthopaedics, The 83nd Group Army Hospital of the People’s Liberation Army (PLA 371 Central Hospital), Xinxiang Medical College, 210 Wenhua Street, Hongqi district, Xinxiang, 453000 Henan China; 3 War Wounded Medical Service Research Office (Department of War Injury and Rescue Service), Army Specialty Medical Center of the People’s Liberation Army (Daping Hospital, Third Military Medical University), Chongqing, 400042 China; 4 Department of Orthopaedics, The 72nd Group Army Hospital of the People’s Liberation Army, Huzhou University, Huzhou, 313000 Zhejiang China

**Keywords:** Assessment, Trunk muscle, Isokinetic strength, Degenerative spinal deformity, Quality of life, Spinal deformity

## Abstract

**Background:**

A considerable portion of the elderly population are increasingly afflicted by degenerative spinal deformity (DSD), which seriously affects patient health-related quality of life (HRQoL). HRQoL index is used across many studies to show correlations between radio-graphical alignment, disability, and pain in patients with DSD. However, imaged structural deformity represents only one aspect for consideration, namely, the disability effect of DSD. We assessed the isokinetic strength of trunk muscle in patients with degenerative spinal deformity (DSD), and investigated its relationship with HRQoL.

**Methods:**

In total, 38 patients with DSD (DSD group) and 32 healthy individuals (control group) were recruited. Both groups were homogeneous for age, weight, height and body mass index (BMI). Assessments were performed using the isokinetic dynamometer IsoMed-2000; trunk extensor, flexor strength and flexion/extension (F/E) ratios were explored concentrically at speeds of 30°, 60° and 120° per second. The grip strength of both hands was measured using a hand-held dynamometer. Visual analogue scale (VAS) scores, the Oswestry Disability Index (ODI), a Roland-Morris disability questionnaire (RDQ), and a 36-item Short Form Health Survey (SF-36) evaluated patient HRQoL. Correlations between trunk strength and HRQoL were analyzed.

**Results:**

When compared with the control group, the DSD group showed lower trunk extensor strength at three velocity movements, and higher F/E ratios at 60° and 120°/s (*p* < 0.05). Both groups exhibited similar trunk flexor strength and grip strength (*p* > 0.05). In DSD group, trunk extensor strength at 60°/s was negatively associated with ODI and RDQ (*p* < 0.05). A negative relationship between trunk flexor strength at 120°/s and ODI was also recorded (*p* < 0.05). In addition, trunk extensor strength at 60°/s and trunk flexor strength at 120°/s were positively correlated with physical functioning and role-physical scores according to the SF-36 (*p* < 0.05).

**Conclusions:**

We identified isolated trunk extensor myopathy in DSD, which causes an imbalance in trunk muscle strength. Isokinetic trunk extensor strength at 60°/s and trunk flexor strength at 120°/s can predict disability, and decrease physical HRQoL in DSD patients.

## Background

A considerable portion of the elderly population are increasingly afflicted by degenerative spinal deformity (DSD), thanks to aging populations and demographic shifts. Due to spinal stenosis, DSD often leads to radiculopathy and low back pain, which seriously affects patient health-related quality of life (HRQoL) [1]. HRQoL index is used across many studies to show correlations between radio-graphical alignment, disability, and pain in patients with DSD [2–4]. However, imaged structural deformity represents only one aspect in consideration regarding of the disability effect of DSD.

Recent evidence has revealed that increased fat infiltration and decreased muscle volumes in trunk muscles are associated with sagittal malalignment in DSD patients [5], suggesting that trunk muscle dysfunction is related to DSD. Trunk muscles control balance and posture, which are essential for normal functional activities, e.g. walking [6]. Because muscle strength is an important aspect of physical performance and functional assessment, previous studies have revealed associations between trunk extensor muscles and HRQoL in low back pain (LBP) patients [7–9]. However, as we know, it had been performed to evaluate the impact of trunk muscle strength of DSD patients on HRQoL in few studies.

Isokinetic dynamometry is an effective and reliable device that measures torque forces produced by specific action muscle groups [10]. Studies have focused on the isokinetic assessment of trunk muscle functions in healthy subjects [11] and LBP subjects [9], while those on trunk muscle strength assessments in DSD patients are rare. Although trunk muscle mass deteriorates with age, and is sensitive to pathological factors [12], specific changes in trunk muscle strength and their effects on DSD are unclear.

Therefore, we studied isokinetic trunk muscle strength in DSD patients, and compared the data with healthy controls. We also studied correlations between these variables and HRQoL in DSD patients.

## Methods

### Participants

This prospective cross-sectional study consisted of 38 DSD patients (DSD group) recruited from March 2018 to November 2019, at a single hospital facility. The inclusion criteria were: (1) aged > 45 years, (2) Cobb angle > 10° or sagittal vertical axis (SVA) > 5 cm, (3) no associated idiopathic, congenital, developmental or neuromuscular spinal abnormalities and no history of spinal surgery, (4) no serious back pain that could affect maximum force assessments. Thirty two healthy subjects, without degenerative lumbar diseases and > 45 years were recruited from the community during the same period. Exclusion criteria for both DSD patients and healthy subjects were: (1) a history of severe back pain within the previous 3 months; (2) new onset of radiologically verified fractures or extremity injury; (3) received physical therapy, acupuncture or back strength training in the last half year. Patient demographics were recorded; sex, age, height, weight and body mass index (BMI). The study was approved by the Ethical Committee of the First Affiliated Hospital, Third Military Medical University, PLA (People’s Liberation Army) (approval number; KY201853). Informed consent was sought from all participants prior to assessments, and all research activities were in accordance with the principles of the Declaration of Helsinki.

### Isokinetic trunk muscle strength assessments

**Fig. 1:**
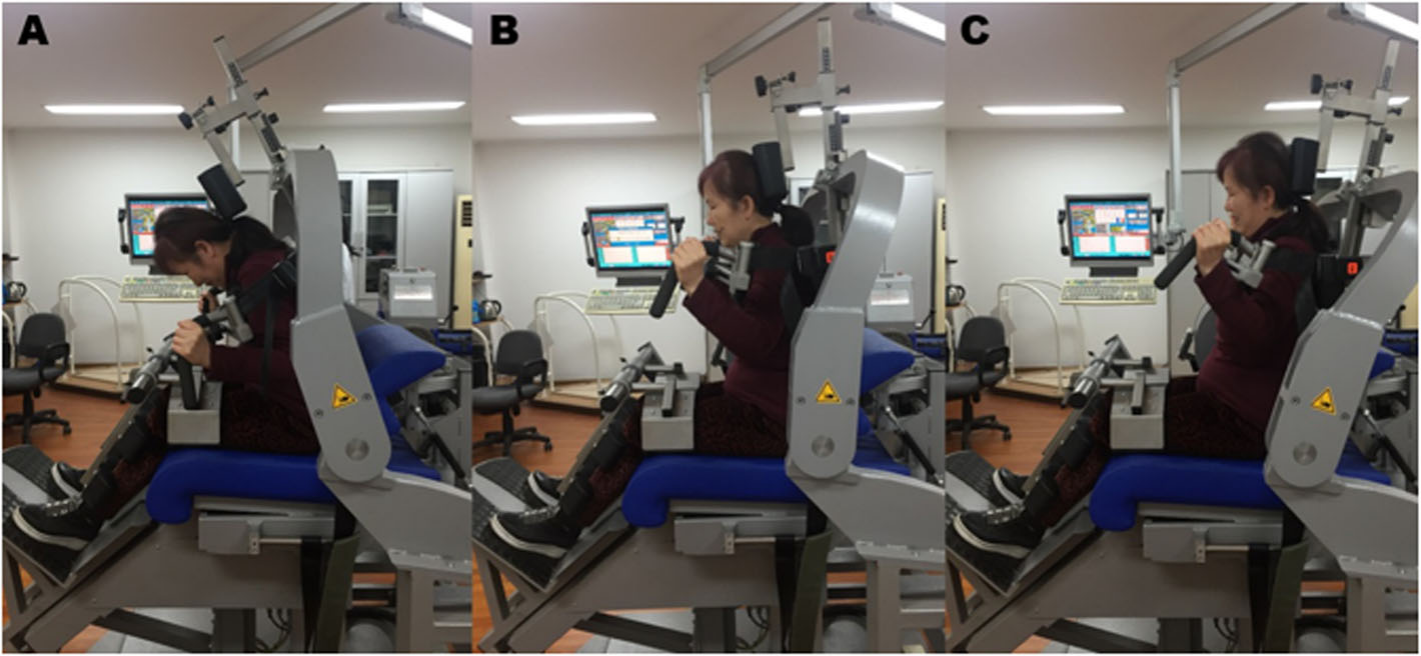
Isokinetic trunk muscle strength test. Illustration: Participant performing a maximum effort of trunk flexion-extension in the isokinetic dynamometer with a range of motion of 40° **a** -20° trunk flexion **b** 0°initial position **c** 20°trunk extension

The trunk muscle strength of all subjects during flexion and extension was measured by isokinetic dynamometer IsoMed 2000 (D&R Technology GmbH Inc., Frankfurt am Main, Germany). Test positions and procedures followed Roth et al. recommendations [13]. The participants were fixed in a sitting position at the shanks, thighs, pelvis and shoulder girdle, with the trunk upright, the hips flexed at 90°, the thighs parallel to the floor to avoid compensatory activation of the lower limbs. Moreover, the location of the dynamometer axis of rotation at the anterior superior iliac spine level and the use of the pad behind the sacrum and the strap on the pelvis minimized hip motion during the protocol. This was considered the initial position. According to Grabiner and Jeziorowski [14], ranges of trunk motion no larger than 50° would isolate lumbar motion, reducing hip flexion–extension. The tested range of motion (ROM) was limited at 40° depending on the movement of the lever arm, this ROM was also recommended in previous study, with 20° (− 20°) of trunk flexion (Fig. 1a) and 20° (+ 20°) of trunk extension (Fig. 1c), relative to the initial position (0°) (Fig. 1b). Concentric exploration of trunk flexors and extensors in three trials’ of five consecutive flexion-extension movements were performed at speeds of 30°/s, 60°/s and 120°/s, as previously used and recommended [15]. Different angular velocities could reflect the various types of muscle contraction and facilitate the understanding of the muscular dynamics of the trunk. The low speed tests examine muscle strength and explosive force, the type of muscle fibers involved in contraction was mainly type I slow contraction muscle fibers. The rapid muscle strength tests assess muscle power and endurance, the contraction type of muscle fibers was mainly type II fast contraction muscle fibers. At medium speed, the major muscle contractile fibers transition from type I to type II [16]. The speed of movement and the execution sequence were set by a computer system of the isokinetic instrument in advance. Once positioned, the speed of movement is constant and the resistance is variable, no matter how much force the subject uses, the speed of body movement will not exceed the pre-set speed. After familiarizing themselves with one or two submaximal practices, each participant completed three trials with five repetitions for the isokinetic mode, starting with the trunk flexion and a subsequent trunk extension sweeping from − 20° to 20°, with regard to the initial position. Participants warmed up 20 to 30 min on a cycle ergometer before testing. All trials were performed with maximal voluntary effort and 1-min break between trials. Verbal encouragements was provided throughout the program to encourage maximum effort. The PT (peak torque) of trunk extensor, flexor and F/E ratios were recorded for each trials.

### Grip strength evaluation

We used a digital dynamometer (CAMRY EH101; Hengqi, Guangdong, China) to assess grip strength. In a standard procedure recommended by the American Association of Hand Therapists, participants sat with their elbows bent at 90° holding a dynamometer, with the meter indicator facing outward, away from the body [17]. Participants held the meter firmly for at least 2 s. One practice test was performed with each hand, and then alternated three times between hands, always starting with the dominant hand. Participants were given verbal encouragement to ensure maximum effort. Maximal values were recorded on the display of the instrument.

### Health-related quality of life assessment

We used visual analogue scale (VAS) scores to evaluate the degree of LBP [18]. The VAS score scale ranged from 0 to 10, the higher the score, the greater the pain intensity. Dysfunction was assessed by the Oswestry Disability Index (ODI) and Roland-Morris Disability Questionnaires (RDQ). ODI scales ranged from 0 to 100%, with a higher ODI indicating more severe dysfunction [19]. RDQ scores ranged from 0 (no disability) to 24 (severe disability) [20]. The 36-item Short Form Health Survey (SF-36) evaluated general quality of life [21], and included nine subscales, comprising; physical functioning (PF), role physical (RP), bodily pain (BP), general health (GH), vitality (VT), social functioning (SF), role emotional (RE), mental health (MH) and health transition (HT). Scores were directly converted to a 0–100 point range. Total SF-36 scores ranged from 0 to 900, the higher the score, the better the physical condition.

### Statistical analyses

All data were expressed as the mean plus standard deviation (SD). Differences between DSD and healthy groups were determined by an independent sample T-test. Comparisons of gender distribution between groups was performed by the Chi-square test. The difference degree (Diff %) of trunk muscle strength between groups was measured using the following formula: Diff % = (high value - low value)/high value × 100%. To explain changes in HRQoL in terms of changes in trunk muscle strength, the determination coefficient, R2 was adopted as an evaluation index. The closer R2 was to 1, the closer the relationship between them. We used Pearson’s correlation coefficients to assess correlations between all evaluated variables. Statistical significance was determined at *p* < 0.05, using SPSS statistical software, version 20.0 (SPSS Inc., Chicago, USA).

## Results

### General information

The mean demographic characteristics of both groups are shown (Table 1). There were no significant differences between groups (*p* > 0.05). In the DSD group, there were 23 cases of degenerative scoliosis, five of degenerative kyphosis and 10 of degenerative scoliosis-kyphosis.

**Table 1:** Demographic characteristics of both groups (mean ± SD)

Variable	DSD group	Control group	Statistics	*P* value
Male/female	8/30	4/28	χ^2^ = 0.394	0.530
Age (years)	63.8 ± 8.0	60.8 ± 6.8	t = 1.675	0.098
Height (cm)	153.4 ± 7.6	153.7 ± 6.9	t = − 0.208	0.836
Weight (kg)	57.3 ± 7.6	57.0 ± 6.2	t = 0.158	0.875
BMI (kg/m^2^)	24.3 ± 2.4	24.2 ± 2.9	t = 0.210	0.835

No significant differences between groups (*p* > 0.05)

*Abbreviations*: *SD* Standard deviation, *DSD* Degenerative spinal deformity, *BMI* Body mass index

### Differences between groups

**Fig. 2:**
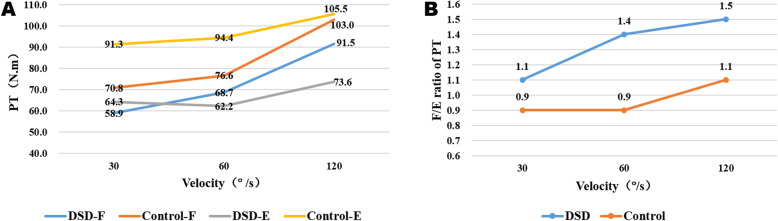
The velocity-changing trend diagram of isokinetic strength of trunk muscle. Illustration: **a** The trunk flexor and extensor PT in the control group were always higher than that in the DSD group, the trends of flexor PT growth of two groups were basically the same and the extensor PT in the control group maintained an increasing trend, while there is a mild downturn for extensor PT from the speed of 30°/s to 60°/s in the DSD group. **b** The F/E ratio in the control group was stable between 0.9 and 1.1, while the F/E ratio maintained an increasing trend from 1.1 to 1.5 in DSD group. PT: peak torque; DSD: degenerative spinal deformity; F: flexion; E, extension

In our study, all patients completed the trunk isokinetic test and grip strength test successfully. Trunk extensor PT values at three velocities were significantly lower in the DSD group, when compared with the control group (*p* < 0.05). Both groups exhibited similar trunk flexor PT values and grip strength in both hands (*p* > 0.05). In addition, F/E at 60°/s and 120°/s speeds in the DSD group were significantly higher than the control group (*p* < 0.05). Differences in trunk extensor PT and F/E degrees between groups were greatest when the velocity was 60°/s (Table 2). The velocity-changing trend diagram of isokinetic strength of trunk muscle showed that both trunk flexor and extensor PT in the control group were always higher than the DSD group. The trunk flexor PT in both groups increased with the increase of angular velocity and the extensor PT in control group also maintained an increasing trend, while there is a mild downturn for extensor PT from the speed of 30°/s to 60°/s in DSD group (Fig. 2a). The F/E ratio of trunk PT in the DSD group was always greater than 1 and kept increasing, while the F/E ratio was approximately 1 and remained relatively stable in the control group (Fig. 2b).

**Table 2:** Trunk muscle strength and grip strength of both Groups (mean ± SD)

Characteristic	30°/s			60°/s			120°/s			GS-left (Kg)	GS-right (Kg)
	Flexor PT (N·m)	Extensor PT (N·m)	F/E	Flexor PT (N·m)	Extensor PT (N·m)	F/E	Flexor PT (N·m)	Extensor PT (N·m)	F/E		
DSD group	58.9 ± 36.3	64.3 ± 48.5	1.1 ± 0.6	68.7 ± 30.8	62.2 ± 39.1	1.4 ± 0.7	91.5 ± 45.9	73.6 ± 46.4	1.5 ± 0.8	24.4 ± 7.5	25.1 ± 8.3
Control group	70.8 ± 24.4	91.3 ± 36.1	0.9 ± 0.4	76.6 ± 25.4	94.4 ± 40.2	0.9 ± 0.4	103.0 ± 40.5	105.5 ± 42.0	1.1 ± 0.5	25.5 ± 5.2	26.0 ± 4.9
Diff %	16.8	29.6	22.2	10.3	34.1	55.6	11.2	30.2	36.4	4.3	3.5
t	−1.574	−2.602	1.912	−1.162	−3.399	3.242	−1.094	−2.995	2.591	−0.656	− 0.537
*P*	0.120	0.011*	0.061	0.249	0.001**	0.002**	0.278	0.004**	0.012*	0.514	0.593

*Abbreviations*: *SD* Standard deviation, *DSD* Degenerative spinal deformity, *PT* Peak torque, *Diff %* Between-group difference, *F/E* Flexion/Extension ratio

*P < 0.05, **P < 0.01, matched analysis

### Analysis of HRQoL associated factors

To minimize the impact of individual differences on the strength of the DSD patient’s trunk muscles, PT was expressed relative to BW (body weight). Using Pearson correlation analysis, the PT/BW of trunk extensor at 60°/s was negatively associated with ODI and RDQ (*R* = − 0.342 and − 0.353, *p* < 0.05, respectively). A negative relationship was determined between the PT/BW of trunk flexor at 120°/s and ODI (*R* = − 0.346, *p* < 0.05), but no significant correlations were observed between F/E ratios and HRQoL (*p* > 0.05) (Table 3).

**Table 3:** Correlation analysis (adjusted body weight) between trunk muscle strength at different velocities and HQOL in DSD patients

R	VAS	ODI	RDQ	SF-36
30°/s Flexor PT/ BW (N·m·Kg^−1^)	−0.109	− 0.129	− 0.205	0.091
30°/s Extensor PT/ BW (N·m·Kg^−1^)	− 0.115	− 0.176	−0.240	0.121
30°/s F/E	−0.131	0.009	0.126	−0.067
60°/s Flexor PT/ BW (N·m·Kg^−1^)	−0.099	−0.281	− 0.279	0.135
60°/s Extensor PT/ BW (N·m·Kg^−1^)	−0.111	− 0.342^*^	−0.353^*^	0.161
60°/s F/E	−0.012	−0.012	0.047	−0.058
120°/s Flexor PT/ BW (N·m·Kg^−1^)	−0.164	− 0.346^*^	−0.271	0.140
120°/s Extensor PT/ BW (N·m·Kg^−1^)	−0.023	−0.145	− 0.189	0.058
120°/s F/E	−0.094	− 0.137	0.010	− 0.013

*Abbreviations*: *SD* Standard deviation, *DSD* Degenerative spinal deformity, *PT* Peak torque, *BW* Body weight, *F/E* Flexion/Extension ratio, *VAS* Visual analogue scale, *ODI* Oswestry Disability Index, *RDQ* Roland-Morris Disability Questionnaires, *SF-36* 36-item Short Form Health Survey

* *P*<0.05, matched analysis

**Fig. 3:**
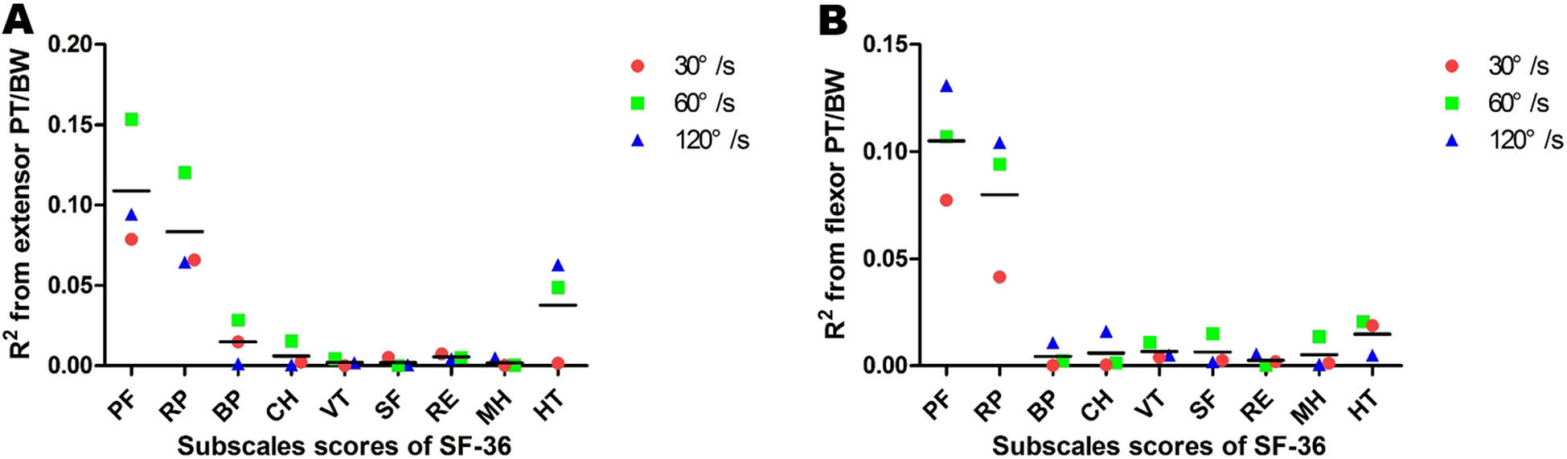
The scatter plot of determination coefficient from PT/BW of trunk muscle on subscales of SF-36. Illustration: **a** The PF and RP were the index most affected by the PT/BW of trunk extensor, especially at the speed of 60°/s. The horizontal line means the average R2 value from extensor PT/BW on subscale scores of SF-36. **b** The PF and RP were the indicators of the greatest influence of the PT/BW of trunk flexor, especially at the speed of 120°/s. The horizontal line means the average R2 value from flexor PT/BW on subscale scores of SF-36. PT: peak torque; BW: body weight; SF-36: 36-item Short Form Health Survey; PF: physical functioning; RP: role physical; BP, bodily pain; GH, general health; VT, vitality; SF, social functioning; RE, role emotional; MH, mental health; HT, health transition

According to correlation analyses between trunk muscle strength and subscale SF-36 scores in DSD patients, the PT/BW of trunk extensor at 60°/s was positively correlated with physical functioning (PF) and role physical (RP) scores (*R* = 0.392 and 0.347, *p* < 0.05, respectively). In determining coefficients, the scatter diagram suggested that in all SF-36 subscales, PF and RP were the two indices most affected by trunk extensor strength, especially at 60°/s (Fig. 3a). In addition, the PT/BW of trunk flexor at 60°/s was positively correlated with PF scores (*R* = 0.327, *p* < 0.05). We also observed significant correlations between the PT/BW of trunk flexor at 120°/s, and PF and RP scores (*R* = 0.362 and 0.323, *p* < 0.05, respectively). The determination coefficient scatter diagram also suggested that PF and RP were key influential indicators of trunk flexor strength at 120°/s (Fig. 3b).

## Discussion

DSD is a common cause of disability and pain in the elderly [1]. Previous studies have highlighted the importance of the sagittal spine for pain and patient quality of life [2–4]. Trunk muscles have an important role in maintaining normal vertebral alignment and the stability of the spine [5]. Therefore, trunk muscles involved in lumbar-stabilization are at the forefront of research needs. Preliminary studies have investigated trunk muscles using histological analyses [22], electromyography [23], ultrasound [24], computed tomography scanning [25] or magnetic resonance imaging [26]. However, trunk muscle strength in DSD patients is an unknown area. Several devices have been developed to assess trunk strength, however the isokinetic dynamometer (IKD) is the gold standard [27]. Trunk flexion and extension force tests are typically performed in the sagittal plane. Isokinetic trunk strength assessments in flexion and extension, using the IsoMed-2000 dynamometer, are highly reliable according to Ralf et al. [13], therefore IKD is ideal for assessing trunk strength in DSD patients.

Our study observed that the DSD group exhibited lower trunk extensor PT, at all three velocities, when compared with the control group, although both groups were undifferentiated in terms of general condition, suggesting the trunk extensor muscle was compromised. Trunk extensor muscle mainly comprises multifidus and erector spinae, which are sensitive to pathological changes [28]. Several studies have shown extensor muscle degeneration in DSD patients; Shafaq et al. demonstrated significant smaller cross-sectional areas of multifidus in patients with degenerative lumbar scoliosis (DLS), when compared to those with degenerative lumbar stenosis (LSS) [22]. Hyun et al. [29] observed that fat infiltration of the multifidus and erector spinae muscles in degenerative lumbar kyphosis patients was significantly higher than in healthy controls. Since back muscle radiological parameters are one of the most valuable indices for predicting back muscle strength [30], it is reasonable to speculate that a decreased size and increased fatty infiltration of trunk extensor muscle, may be associated with decreased trunk extensor isokinetic strength. Additionally, skeletal muscles tend to suffer with ‘disuse atrophy’ with lower activity levels and reduced muscle strength requirements [31]. DSD patients are often reluctant to perform trunk extension and strength training due to back pain, resulting in ‘disuse atrophy’ of trunk extensor muscles, culminating in a decline in muscle fiber recruitment. Thus, the ability to generate muscle strength is reduced, resulting in weakness of the trunk extensor.

Our findings showed that both groups exhibited similar trunk flexor and grip strength, suggesting the trunk extensor muscle is impaired exclusively in DSD patients. Yaji et al. [32] observed that muscular degeneration of the trunk extensor in DLS patients while the muscle strength and volume of the other body parts were normal, indicating that local myopathy rather than total degenerative loss of skeletal muscle which was called sarcopenia. A similar observation was recorded in lumbar degenerative kyphosis (LDK) patients [26]. Our study confirmed and extended the previous studies through muscle strength level, although the cause and effect relationship is still controversial.

Trunk extensor and flexor muscles interact with each other to maintain biomechanical stability of the lumbar spine [33], therefore evaluating the balance of trunk flexor, extensor muscle strength is of great significance, and the F/E ratio is an important evaluation index [34]. Spinal muscle balance is beneficial for F/E ratios < 1 in terms of equilibrium [34]. In this study, the F/E ratio of the control group was 0.9–1.1, which was within the ratio range of a normal population, whereas the F/E ratio in the DSD group, at 30°/s, 60°/s and 120°/s, were 1.1, 1.4 and 1.5, respectively. In addition, we observed higher F/E ratios at 60°/s and 120°/s, when compared with the control group, suggesting an imbalance in trunk flexor and extensor muscle strength in DSD patients. Although the PT of trunk flexor and extensor muscles in the DSD group was always lower than the control group, only extensor PT exhibited significant differences between groups. Therefore, we propose that impairments in trunk extensor muscles causes an imbalance of trunk muscle strength in DSD patients, and this imbalance is identified at speeds of 60°/s and 120°/s (fast) in isokinetic assessments.

Regular training of trunk muscle, and good core strength are important for daily life and physical activities [35]. Therefore, it is important to clarify the impact of trunk muscle strength changes on the quality of life of DSD patients. A previous study revealed that maximal muscle strength was observed in patients with higher body weight [36]. Granito et al. [37] also pointed out that the peak torque should be normalized according to body weight before analyze the correlations between age and peak concentric and eccentric torque of the trunk flexors and extensors. Therefore, we adopted relative PT (N·m·Kg^− 1^) as the index, while accounting for body weight, to minimize the influence of individual differences on correlation analyses between trunk muscle strength and quality of life of DSD patients.

Our study revealed significant correlations between trunk extensor PT at 60°/s and disability scores, including ODI and RMQ scores. This suggested that decreased strength in the trunk extensor muscle reflected increased disability in DSD patients, consistent with previous reports. Keller et al. [38] observed that the correlation coefficient between trunk extensor PT at 60°/s and ODI was − 0.57 in LBP patients. Kudo et al. [39] suggested that when compared with the sagittal position of spinal and lower limbs, trunk extensor strength was the most reliable index of RDQ scores in the elderly. Seo et al. [36] showed that trunk extensor strength is negatively correlated with ODI score. A significant relationship was observed between trunk flexor PT at 120°/s and ODI scores in our study. Vieira et al. [35] showed that the strength of abdominal muscles in elderly patients with lumbar osteoarthritis, was directly proportional to their quality of life. Based on current data, we propose that both extensor and flexor muscles of the trunk are important for quality of life, while extensor muscle strength may be more important.

We also found that both trunk extensor and flexor strength predicted physical functioning and role-physical scores. Previous studies have shown that weakness in trunk muscle strength in the elderly, leads to increased fall tendencies, impaired mobility, impaired daily living activities and increased disability [40]. While strong trunk muscles can not only decrease the kyphotic, but also accelerate the recovery of normal physical activities [41]. Thus, trunk muscle strengthening should be considered a specific intervention in preventing spinal deformity.

Our data showed that isokinetic trunk extensor strength at 60°/s and trunk flexor strength at 120°/s predicted patient HRQoL. This discrepancy in velocity may be related to variant muscle fiber recruitment, and pathological deterioration in strength at different speeds [15]. We propose that muscle contraction intensity of trunk extensor and flexor muscles coincide with the set compliant resistance of IKD at 60°/s and 120°/s, respectively. Therefore, it better reflects the true level of muscle and its impact on patient quality of life.

Both muscular strength and endurance are important evaluation indexes reflecting the function of the trunk muscle. We did not test trunk muscle endurance in this study for the following three reasons. First, the subjects were generally older, some of whom had osteoporosis and varying degrees of lower back pain. These subjects were prone to fatigue during the test, and continuous exercise may increase the risk of injury. Second, the use of isokinetic measures seems to be common in assessing maximum trunk strength capacity but extremely rare for the quantification of local muscular endurance [42]. Third, the static endurance of isometric model is a better evaluation of trunk muscle endurance in patients with low back pain than dynamic endurance of isokinetic model. Gruther et al. [43] pointed that the isokinetic trunk endurance test appears to be problematic because of learning effects and recommend the Biering-Sørensen test for management of chronic low back pain rehabilitation. As we did not evaluate the association between trunk muscle isometric endurance and HRQoL in present study, so further research is required in this area.

Our study had some limitations. Firstly, participants were recruited from a single center, and the sample size was relatively small. However, this was the first study to report isokinetic trunk muscle strength in patients with degenerative spinal deformity, and its association with HRQoL. Secondly, DSD is often combined with degenerative discs, endplate degeneration and other degeneration, however these factors were not considered here, and thus may affect some quality of life, potentially introducing some bias to our data. Thirdly, there were fewer males, and primarily scoliosis deformities in our sample. Therefore, in future studies, we will expand sample size and make comparisons between patients of different genders and deformities.

## Conclusions

We showed that isolated trunk extensor myopathy in DSD causes an imbalance in trunk muscle strength. In addition, isokinetic trunk extensor strength at 60°/s and trunk flexor strength at 120°/s predicts disability and physical HRQoL in these patients. These preliminary data may provide a clinical intervention strategy to improve trunk function, thus improving DSD patient HRQoL.

## Data Availability

The datasets used and/or analysed during the current study are available from the corresponding author on reasonable request.

## Abbreviations

DSD: Degenerative spinal deformity
HRQoL: Health-related quality of life
BMI: Body mass index
F/E: Flexion/extension
VAS: Visual analogue scale
ODI: Oswestry Disability Index
RDQ: Roland-Morris disability questionnaire
SF-36: 36-item Short Form Health Survey
